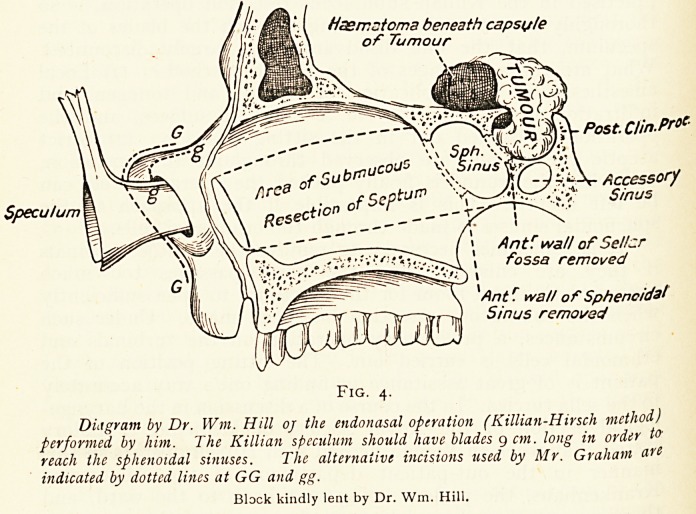# Otology and Rhinology

**Published:** 1913-12

**Authors:** P. Watson-Williams


### OTOLOGY AND RHINOLOGY.

**Direct Salpingoscopy.**—The naso-pharyngoscope devised by Holmes was at first received as an interesting method for obtaining nearly as good a view of the same parts that can be brought under inspection by the posterior rhinoscopic mirror and the laryngoscope. But it is because we are enabled to inspect regions in the nasal passages and in the nasopharynx that cannot be seen by any other means that rhinologists have learnt to value the instrument of Holmes and others of similar pattern, which may well be likened to a very fine calibred cystoscope. And although much of the Eustachian orifice and the region around can be viewed by indirect rhinoscopy, with direct endo-rhinoscopy one certainly gets not only a different view, but often a much better view of the actual orifice of the tube, as well as of its anterior and posterior lips. Walker Wood has recorded an analysis of the nasopharyngeal findings in about 650 cases examined, and his results are instructive and valuable. There were forty abnormalities, in fifteen the Eustachian tubes being bifid, and in three there were adventitious openings. He figures cases of mucous polypus in the mouth of the tube, fibroma of the cushion, and a postnasal polypus; and these illustrations the author has permitted us to reproduce. In other of his figures one finds examples of adenoid remains in Rosenmüller fossa, and bands extending across the fossa, due to untreated adenoids and several varieties of salpingitis. As regards acute salpingitis, the author states that "this is, perhaps, the most important condition affecting the Eustachian tube, as its resulting effects are so grave. I have carefully inquired into many hundreds of cases of chronic middle-ear catarrh, and in a very large proportion I have found that these patients suffered from attachs of ear-pain or ear-ache in childhood. These were attacks of acute or subacute middle-ear

Three examples of inflammatory conditions of the Eustachian Tube, as they appear on direct examination with the naso-pharyngoscope.

WALKER WOOD.

FIG. 1.

*Mucous polypus in the mouth of the tube (right).*

FIG. 2.

*Fibroma of cushion. Adenoid granules in Rosenmüller's Fossa.*

FIG. 3.

*Postnasal polypus.*

We are indebted to the Author, and to Messrs. Adlard & Son, for the loan of these blocks from the original article here abstracted.—ED.

catarrh, and salpingitis arising from adenoids, bad teeth, or chronically-inflamed tonsils." The symptoms of acute salpingitis are (1) pain in neck or ear, almost always ear-ache, (2) deafness, (3) tinnitus, (4) drum injected and red, (5) mouth of Eustachian tube is red, glazed and swollen, and there may be a slight exudate of mucus from the mouth. The swelling is confined to the mouth and the tissues immediately around it, and is not a general inflammation as in chronic salpingitis, while the minute vessels seen in health, and which are often engorged in chronic conditions, become obscured in acute conditions.

Malignant growths may arise at the mouth of the Eustachian tube, and the author states that "probably they are much more common than is supposed," and in early doubtful cases the naso-pharyngoscope enables the surgeon to remove a fragment for microscopical investigation with great precision.

**The Operative Treatment of Tumours and Disease of the Pituitary Body** was the subject of an instructive discussion in the Laryngological Section of the recent International Congress of Medicine held in London, and has also been pressed on our notice by many recent contributions and reported cases, and more particularly by the published work of Harvey Cushing, and a discussion on Diseases of the Pituitary Body at the Royal Society of Medicine. Schäfer, in his contribution on the structure and function of the pituitary body, states that this body is developed partly from the ectoderm of the buccal cavity, partly from the neural canal, *i.e.* it is partly epithelial and partly nervous in origin, the former comprising two portions, the *pars anterior* and the *pars intermedia.* The anterior lobe appears to be related to the general growth of the body, the posterior lobe, which includes the *pars intermedia,* probably influences the contractility of plain muscle fibres, and excites activity in the kidney and mammary gland. With regard to operations on the pituitary body, it is recognised that the hypophysis can be approached either by the intra-cranial route or through the sphenoidal sinus (the extra-cranial route), and it is the latter which we are concerned with in rhinology. Though first suggested by Giordano in 1897, the trans-sphenoidal operation was first performed by Schloffer in 1907, when he successfully removed an hypophyseal adenoma. Various routes to reach the sphenoidal sinus have been adopted, viz. (*a*) the palatine route, *i.e.* by dividing the soft palate, and removing first the horizontal plate of the palate bones, and secondly the corresponding posterior portion of the vomer. This route was adopted twice by Durante and by Ballance in 1909. (*b*) By various operations involving external division of the nose, *e.g.* Ollier's operation, turning the nose up, Von Brun's operation, or Moure's operation, lateral rhinotomy, and Kanavel's method of turning the nose up (inverted Ollier operation), and Dialti's method of splitting the nose in the middle line, and turning the two flaps outwards. By whichever method the nasal passages are laid open, the septum has to be largely removed, and followed by the exenteration of the ethmoid cells, and the middle and inferior turbinals. (*c*) Endonasal routes. Of these the only two methods that have been used in a considerable number of patients are (1) the Halsted operation suggested by Löwe, and first performed by Halsted, and later adopted by Cushing, and (2) the Hirsch method. The Halsted operation begins with a transverse sublabial incision, as in the Rouge antral operation, followed by submucous resection of the septum, and carried out after upward retraction of the upper lip.

Hirsch first carries out an ordinary Killian resection of the septum right back to the anterior wall of the sphenoidal sinuses, then widely separating the two layers of mucous membrane by strong, long Killian dilating forceps, he crushes the middle turbinals against the outer walls of the nasal passages, and thus exposes to view the anterior walls of the sphenoidal sinuses. The anterior walls of these sinuses are then removed, and with bone-cutting forceps the sphenoidal sinus septum is removed, thus exposing to view the posterior wall of both sinuses. A transverse incision is made in the anterior wall of the sella turcica by means of a chisel, then with a special hook-shaped elevator inserted between the bone and dura, follows, the removal of this portion of the posterior sinus wall corresponding to the pituitary fossa, and then with a nibbling bone forceps the dura is exposed over as large an area as feasible, and a flap of the dura turned down. The exposed tumour is then pierced, to ascertain whether it is cystic or solid. If the former, as much of the cyst wall is excised as possible; if solid, it is removed with a curette, working mainly from above downwards. Finally, a strip of iodoform gauze is inserted between the muco-periosteal flaps. The disadvantage of the Hirsch operation lies in the distance to be traversed before reaching the tumour, and the necessity of working through the somewhat narrow passage between the blades of the long Killian speculum. On the other hand, there are many advantages in this over all the methods which involve external rhinotomy, besides which every rhinologist being practised in the Killian submucous resection operation, is so thoroughly accustomed to working through the blades of the speculum, that the one disadvantage is largely discounted. What are the advantages of the Hirsch method? (1) Local anæsthesia by the application of cocaine and tonogen, and infiltration with Schleich's No. 2 solution suffices, and the operation is carried out in the sitting position. (2) Strict aseptic precautions are observed throughout the operation, and when the wound is finally packed the operative field can be left aseptic. This is impossible if the approach to the sphenoidal sinuses is made through the ethmoidal cells.

It is sometimes necessary to remove the middle turbinals if they are enlarged, and the nasal passages too much narrowed to afford room for the speculum to open sufficiently when the submucous resection has been made. Under such circumstances, a preliminary operation on the turbinals and ethmoidal cells is carried out. The sitting position of the patient is of great asssitance in finding one's way accurately to the sella turcica. In the course of a discussion in the Laryngological Section of the Royal Society of Medicine, O'Mallory stated that when in Vienna he had seen Hirsch operate in this manner in the out-patient department of the Allgemeine Krankenhaus, the patient being sent back to the ward, and there was no more hemorrhage in the course of the operation than one usually sees in a submucous resection.

Hirsch in his communication to the International Congress gave statistical results on thirty-five cases in which he had operated. In his first twenty-five cases reported previously he had three deaths, giving a mortality of 11.5 per cent., and with two other cases successfully operated on by Spiess and Holmgrew by the septal route the mortality was reduced to 10.7 per cent. The report of his later results are not yet available.

Two cases operated on by British rhinologists by the endonasal route have recently been placed on record. Graham operated under intravenous ether anæsthesia in 1912, making an incision in the mid-line from the tip of the nose to the upper lip, so as to divide the columella and expose the free edge of the septal cartilage, and then a Killian septal resection was performed, and the operation completed in the manner described as Hirsch's operation. The patient left the hospital very much relieved twenty days after the operation. A second operation was performed two months later, the approach to the sphenoidal sinus being made through the left nasal fossa proper, as it was felt that there would be difficulty in separating the septal flaps of the previous operation, but the patient died suddenly, collapsed thirty hours after the operation. Hill operated on a

FIG. 4.

*Diagram by Dr. Wm. Hill of the endonasal operation (Killian-Hirsch method) performed by him. The Killian speculum should have blades 9 cm. long in order to reach the sphenoidal sinuses. The alternative incisions used by Mr. Graham are indicated by dotted lines at GG and gg.*

Block kindly lent by Dr. Wm. Hill.

patient by Graham's modified Hirsch method, but she died from hemorrhage eight hours later. The case was found *post-mortem* to have been unsuitable for operation, but there was no means of ascertaining this beforehand. In both these cases the misery of the patient's condition before operation was such as to justify the grave risk which must always attend any operation on the pituitary body, but such operations cannot be commended in cases which do not present grave symptoms.

REFERENCES.

Walker Wood, *J. Laryngol.*, 1913, xxviii. 571.

Hill, "The Operative Treatment of Pituitary Tumours," *J. Laryngol.*, 1913, xxviii. 337.

"Discussion on Diseases of the Pituitary Body," Royal Society of Medicine, *J. Laryngol.*, 1913, xxviii. 354.

"Report of Proceedings of Laryngological Section of Royal Society of Medicine," *Ibid.*, p. 369.

Hirsch, "The Operative Treatment of Tumours of the Hypophysis Cerebri by Endonasal Methods," *Arch. f. Laryngol.*, xxvi. pt. 3; abstract in *J. Laryngol.*, 1913, xxviii. 378.

Harvey Cushing, "The Pituitary Body and its Disorders," 1913; Review in *J. Laryngol.*, 1913, xxviii. 382.

Jules Broeckaert, "A Contribution to the Surgery of the Hypophysis," *J. Laryngol.*, 1913, xxviii. 340.

P. WATSON-WILLIAMS.

---

## Figures and Tables

**Fig. 1. Fig. 2 Fig. 3. f1:**
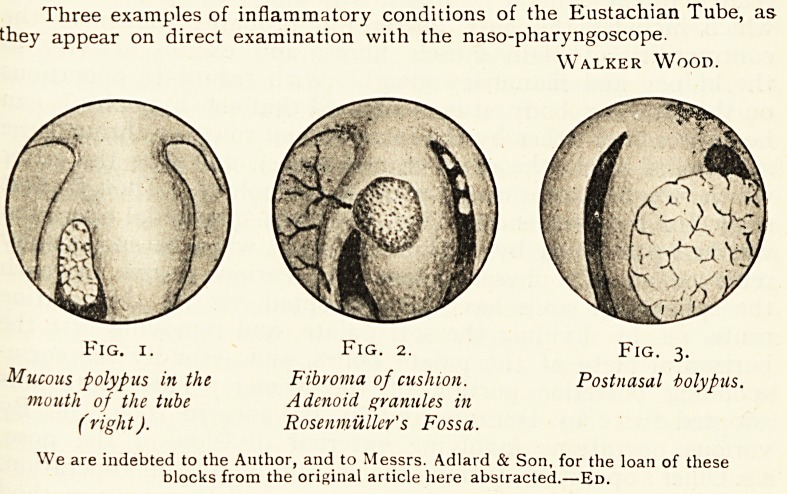


**Fig. 4. f2:**